# Mothers’ feeding practices among infants (4–12 months) and associated factors: a cross-sectional study in Saudi Arabia

**DOI:** 10.1017/jns.2022.85

**Published:** 2022-09-23

**Authors:** Salwa Ali Albar

**Affiliations:** Food and Nutrition Department, King Abdulaziz University, Jeddah, Saudi Arabia

**Keywords:** Mothers’ feeding practices, Breast-feeding, Infants health care, Weaning practice, Saudi infants

## Abstract

A global target of increasing exclusive breast-feeding (EBF) to at least 50 % by the year 2025 was set by the WHO for infants under 6 months. The lowest prevalence in the world was found in the Eastern Mediterranean region in 2010–18 and little is known about the status of mothers’ feeding practices in Saudi Arabia. The present study aimed to assess mothers’ actual feeding and weaning practices used with their infants by the mothers’ different age groups. The present study was conducted among 247 mothers of infants aged 4–12 months who were attending public well-baby clinics. Quantitative data were obtained by nutritionists using an electronic semi-structured questionnaire about mothers’ feeding practices. Only 5·3 % of mothers engaged in EBF, 44·9 % breast-fed their infants after an hour of birth, while 92·7 % of infants had ever been breast-fed. The average intent/plan to continue breast-feeding was 4·9(±3·1) months. Younger mothers introduced weaning food around 4 weeks earlier than older mothers (mean differences were −0·4, 95 % CI −0·71, −0·13; *P* = 0·031). A total of 64·3 % of infants received complementary feeding before completing 17 weeks. Maternal age group and delivery mode were the only factors associated with the early introduction of complementary feeding. A total of 69·2 % of the mothers believed that ‘it is a good time’ and 61·1 % felt that ‘infants are hungry and need other sources of food’. Online sources and family advice were the top sources of information on mothers’ feeding practices. Provision of professional advice about EBF and optimal weaning practices are significant areas for improvement in terms of compliance with recommended infant feeding practices.

## Introduction

Good infant feeding practices, in particular breast-feeding, are considered the most effective way to ensure a child's health and survival. The World Health Organization (WHO) and United Nations (UN) recommend that mothers in developing and developed countries should exclusively breast-feed (EBF) their infants for the first 6 months (24 weeks) of their lives^(1)^, and then ensure that they receive adequately safe and nutritious solid foods about two to three times a day at age 6–8 months, increasing this to three to four times daily at age 9–11 months, with continuous breast-feeding until they reach 2 years old or older^(2,3)^. Additional healthy snacks should be offered one to two times/day by age 12–24 months^(1)^. However, over the period 2015–2020, only 40 % of infants were exclusively breast-fed^(4)^. Nearly two out of three infants are not exclusively breast-fed and this rate has not been enhanced in the last two decades^(4)^. Current practices in the Middle East do not adhere to WHO recommendations. For instance, in Lebanon, United Arab Emirates, and Iraq, 53, 70 and 79 % of infants, respectively, are given complementary feeding between the ages of 4 and 6 months^(5)^. Available information indicates that feeding practices are not optimal among Saudi infants^(6)^. The prevalence of EBF in Saudi was extremely low, at 1·7 % in 2010, and partial breast-feeding was the most common feeding style^(7)^. While in 2017, the rate of EBF was 31·4 % in North Saudi^(8)^, and the early intention of breast-feeding (EIBF) was fair in the Central, Western and Eastern regions in Saudi^(9)^. One study has shown that 62·5 % of Saudi infants consume complementary feeding before reaching 4 months of age^(10)^.

The way in which infants are fed in their first year of life has important consequences in terms of the short- and long-term impact on an infant's health^(11)^. A number of studies have shown that infants who are not breast-fed are more likely to develop certain health conditions and diseases, such as lower respiratory tract infection and gastrointestinal infection^(11–14)^.

The gradual introduction of complementary feeding or ‘weaning practice’ is very critical and essential to meet an infant's physiological stage (as their neurodevelopment, gastrointestinal systems and kidneys are still underdeveloped^(15)^) and nutritional requirements in their first year of life^(16)^. As recommended by the WHO, complementary feeding should be given in a timely and appropriate way, beginning at 6 months of age (after 5 months), and at appropriate amounts, frequency, consistency and using a good variety of foods^(3,17)^. An early introduction of solid food has been found to increase the incidence of respiratory infection and allergy^(18)^. Also, it has been associated with an increased risk of eczema, allergies^(19,20)^ and the odds of obesity at 3 years of age^(21)^.

There is some evidence suggesting that weaning infants beyond the recommended time may increase the percentage of body fat in childhood^(22)^. One systematic review suggests that very early introduction (at 4 months) may increase the risk of being obese and overweight in childhood^(23)^, and it may inversely affect dietary patterns and feeding behaviours^(24,25)^. It has also been found that food introduced in the first 24 months predicts young children's dietary intake^(26)^.

Saudi Vision 2030 has contributed to economic, social and cultural developments that have had a positive impact on women's participation in public spheres, improving women's status, empowering them and raising the level of their economic participation. As a result, the percentage of women in the labour force in Saudi Arabia (SA) rose from 24 to 27·5 % in the first quarter of 2020^(27)^. These social improvements may lead to a number of changes in mothers’ breast-feeding and general feeding practices among infants, especially young and first-time mothers. Furthermore, it is expected that the WHO's 2025 global targets in EBF will be disrupted due to the COVID-19 pandemic^(28)^.

In SA, there has been a limited number of studies focusing on breast-feeding practice in general^(7,10)^, as well as on specific regions of SA. However, inadequate information is available in regards to EBF and mothers’ complementary feeding practice^(10)^. Furthermore, a review study in Saudi found that maternal age was an important factor associated with the prevalence of breast-feeding^(29)^. Therefore, the present study aims to investigate Saudi mothers of different age groups and their actual feeding and weaning practices used with their infants (4–12 months), as well as to examine some factors that could influence the mother's decision.

## Materials and methods

### Study population

This is a cross-sectional survey conducted in Jeddah city, SA, between August and November 2021 among 247 mothers of fertility age (18–45 years old) and their infants. The estimated sample size for this research was calculated based on the findings from a previous study that found the prevalence of early introduction of complementary feeding at <17 weeks among infants was 62 %^(10)^, with a 95 % confidence level and a confidence limit of 5 %. The resulting sample size was 230 mothers, which was then approximated to 253 mothers, allowing for a 10 % attrition rate.

To gather data on the mothers’ feeding practices for their current infants aged between 4 and 12 months, the research team visited the pre-specified well-baby clinics (held at the largest public centre in Jeddah and serving a large number of people from different areas and from different socioeconomic statuses). In SA, all children under 5 years old have to visit a well-baby clinic for their infant vaccinations. The research team screened the mothers and their infants based on eligibility criteria, informed them of the objectives and invited them to participate in the study. The recruitment followed convenient sampling. Mothers and infants were chosen based on inclusion criteria. The Saudi mothers were aged between 18 and 45 years old and free of chronic disease. The infants had been of ≥37 weeks gestational age (full-term babies), aged 4–12 months and did not require specialised care. The exclusion criteria were mothers with chronic disease, preterm babies (<37 weeks of gestational age) and infants with congenital disease or a disease that could prevent breast-feeding.

All mothers who were willing to participate in the study were asked to sign a consent form. This study was conducted according to the guidelines laid down in the Declaration of Helsinki and all procedures involving research study participants were approved by the Ethics Committee of King Abdulaziz University, Unit of Biomedical Ethics (reference: 443-21). Written informed consent was obtained from all subjects. A total of 97·6 % of the mothers approached agreed to participate in the study (recruitment rate).

### Data collection

An electronic semi-structured questionnaire was developed using national and international questionnaires^(7,10,30,31)^. The questionnaire was content validated and culturally adapted by a panel of experts consisting of two neonatal physicians and a nutrition epidemiologist. Then the questionnaire was piloted among twenty-seven mothers prior to being administered among the final sample. The original version of the questionnaire was written in English and then translated into Arabic, since the questionnaire was planned to be administered to the study sample in their native Arabic language. The original and translated questionnaires were reviewed for consistency in terms of meaning. The questionnaire was administered by trained nutritionists through face-to-face interviews.

The questionnaire was semi-structured and consisted of three parts covering the mother's past/present breast-feeding and weaning feeding practices, the mother's demographical information, and the mother's general knowledge and sources of information.

#### Mother's feeding practices

Yes or no questions were asked regarding the mother's breast-feeding practice; for example, regarding EBF (whether their baby had only breast-milk from birth), EIBF, and the mother's intention to continue breast-feeding. The definition of EBF was in accordance with the WHO's definition, that the infant receives only breast-milk during the first 6 months of his/her life and no other solids or liquids are introduced^(31)^; feeding of expressed breast-milk counts as being fed breast-milk. Also, mothers’ EIBF was defined as the percentage of infants who are breast-fed within an hour of birth. Furthermore, multiple choice questions were asked regarding the mother's knowledge of the WHO's EBF definition and the importance of breast-feeding infants within an hour of the infant's birth. Also, an open-ended question (based on the number of months) was included in the questionnaire regarding the mother's intention to continue breast-feeding her infant in general (duration), even if she had introduced different feeding practices. Also, a question about whether their baby had ever received breast-feeding (EvBF) was asked.

The early introduction of complementary feeding was defined in this study as the introduction of any food before the infant reaches 17 weeks. This was defined based on the European Society's guidance, that infants younger than 17 weeks should not be given any complementary foods^(32)^, and has been used in many Western^(33,34)^ and Arab countries^(10,35)^ in studies with related objectives^(10)^. Complementary food was defined in this study based on the definition used by the Saudi Food and Drug Authority^(36)^, which is in line with the European Food Safety Authority (EFSA)^(37)^. It includes all foods that are given to infants other than breast-milk, formula-milk, water or vitamins, and can be beverages, spoon-fed pureed foods, spoon-fed lumpy foods or finger foods, either prepared at home or produced commercially. Data on the early introduction of complementary feeding were generated from questions that asked mothers to state at which point in time they introduced complementary food to their infant.

Furthermore, open-ended questions were used to asked about the infant's age by month the first time a breast-milk substitute (formula, fresh or processed animal milk) was introduced, as well as beverages (other than milk and water), infant homemade food, infant ready-to-eat-food and regular family food.

#### Mother's demographical information

The survey collected data on the mother's age, education, occupation status, family income, number of children, current contraceptive and infant delivery mode. The mother's current anthropometric information was self-reported while the infant's anthropometric data were collected from the infant's vaccination record. Mother's body mass index (BMI) was calculated (weight (kg)/height (m^2^)) and the value was interpreted based on WHO classifications, where BMI < 18·5 kg/m^2^ is underweight, 18·5–24·9 kg/m^2^ is normal, 25–29·9 kg/m^2^ is overweight and ≥30 kg/m^2^ is obese^(38)^. Furthermore, the mother's sources of knowledge and reasons behind their practices were examined; the mothers could give more than one answer to ensure a better understanding of their practices.

### Statistical analysis

The data gathered from the surveys were inputted and analysed using Stata statistical software release 12 (Stata Corporation)^(39)^. Descriptive statistics were used to describe the general characteristics for the sample, for continuous variables such as height and weight the mean and ±sd were used, and for categorical variables like the mother's education level, occupation and family income, numbers and proportions were reported. An independent two-sample *t*-test and Pearson Chi-Square test were used to test the differences between younger mothers (18–29 years) and older mothers (30–45 years) in terms of their general characteristics.

Differences between younger mothers and older mothers in terms of their infant feeding practices were assessed using a regression model (with mother's feeding practice as a dependent variable and mother's age group as an independent variable) with adjustment made for the mother's education, occupation, family income, BMI, and the infant's gender and birth weight. In accordance with the literature, these variables were considered risk factors for early weaning practice^(10,40–42)^. Furthermore, a binary logistic regression model, with the introduction of complementary feeding as a dependent variable, was then used to determine the variables that independently predicted early weaning among infants (mother's age group, education, occupation). Findings were assessed by the OR and 95 % CI. A statistical significance level of *P* < 0·05 was used.

## Results

### Sample characteristics

A total of 247 mothers aged between 18 and 45 years old participated in this study, and 59·9 % of them were of a younger age (24·9 ± 2·5). There were significant differences between the older mothers (30–45 years) and younger mothers (18–29 years) in terms of their BMI category. A total of 71 % of mothers had a bachelor's degree and 94·7 % of them did not smoke cigarettes. Most of the mothers came from second or third family income classes, at 48·6 and 23·9 % respectively, and there was no significant difference in this in relation to the mother's age group. A total of 55·5 % of the mothers were housewives and there was a significant difference between younger and older mothers in terms of their occupation status. Older mothers had a significantly greater number of children (2·9 ± 1·5) and family members (5 ± 1·5) compared to younger mothers. More than half of the infants who participated in this study were female (57 %) and there was no significant difference between mothers of different age groups in terms of their infant's weight (Table 1).

**Table 1.** General characteristics of study participants by total sample and mother's age group

General characteristics	All participants (*n* 247)		18–29 years (*n* 148)		30–45 years (*n* 99)		*P*
	Mean	±sd	Mean	±sd	Mean	±sd	
Mother age (years)	28·6	5·4	24·9	2·5	34·0	3·8	
Mother height (cm)^a^	158·2	6·0	158·4	6·0	157·9	6·0	0·572
Mother weight (kg)^a^	63·7	12·8	60·9	12·2	67·9	12·6	0·000
Mother current BMI (kg/m^2^)^a^	26·1	12·6	25·5	15·6	27·2	5·1	0·285
BMI category	*N*	%	*N*	%	*N*	%	*P*^b^
Normal weight	127	51·4	91	61·4	36·3	36·6	0·001
Overweight	78	31·6	41	27·7	37	37·4	
Obese	42	17	16	10·8	26	26·3	
Mother education level^b^							
High school and under	48	19·4	26	17·6	22	22·2	0·119
Bachelor/College	176	71·3	112	75·7	64	64·7	
Postgraduate	23	9·3	10	6·7	13	13·1	
Smoking^b^							
Yes	13	5·3	7	4·7	6	6·1	0·646
Number of cigarettes (yes; *n* 13)^b^							
1–4	9	69·2	5	71·4	3	50·0	0·558
≥5	4	30·8	2	28·6	3	50·0	
Family income (Saudi Riyal)^b^							
≤4·000	42	17·0	30	20·3	12	12·1	0·122
5·000–11·999	120	48·6	73	49·3	47	47·5	
12·000–19·999	59	23·9	33	22·3	26	26·3	
≥20·000	26	10·5	12	8·1	14	14·1	
Mother occupation^b^							
Student	43	17·4	39	26·3	4	4·0	0·000
Employed (full time)	43	17·4	17	11·5	26	26·3	
Employed (part time)	24	9·7	10	6·7	14	14·1	
Housewife	137	55·5	82	55·4	55	55·6	
Current use of contraceptive^b^							
Yes	151	61·1	97	65·5	54	54·5	0·097
Type of contraceptive (yes; *n* 151)^b^							
Pills for BF	36	23·8	17	17·5	19	35·2	0·025
Ordinary pills	34	22·5	28	28·9	6	11·1	
IUD	27	17·9	17	17·5	10	18·5	
Arithmetic	43	28·5	26	26·8	17	31·5	
Implant	11	7·3	9	9·3	2	3·7	
Number of children	2·1	1·3	1·6	0·7	2·9	1·5	0·000
Delivery type^b^							0·973
Vaginal	155	62·8	93	62·8	62	62·6	
Caesarean section	92	37·2	55	37·2	37	37·4	
Infant gender^b^							
Male	106	42·9	67	45·3	39	39·4	0·361
Female	141	57·1	81	54·7	60	60·6	
Infant birth weight (kg)^a^	2·8	0·6	2·8	0·6	2·8	0·6	0·921
Singleton	241	97·6	144	97·3	97	97·9	0·733

a Differences were assessed by using independent sample *t*-test.

b Univariate analyses using cross-tabulation and *χ*^2^ statistical tests to compare the differences between mothers’ different age groups.

### Breast-feeding practice by mother's age group

Only 44·9 % of mothers had EIBF their infants after an hour of birth, and the average number of infants aged 4–12 months who had ever been breast-fed (EvBF) was 92·7 %. However, only 5·3 % of mothers adhered to the WHO definition of EBF of infants aged 6–12 months (this has been calculated by counting the number of infants aged 6–12 months EBF/total number of infants aged 6–12 months, *n* 152). Furthermore, none of the infants aged 4–5 months received EBF. Although mothers of an older age were found to be of a higher proportion than younger mothers in terms of EIBF, EvBF and EBF, there were no significant differences between mothers’ age groups.

Only 2·4 % of mothers believed in the importance of breast-feeding their infant in the first hour of their life, 14·9 % of mothers believed that it has no importance, and 82·6 % do not know about it; there were significant differences between mothers’ age groups (*P* = 0·040). Furthermore, only 10 % of mothers knew the WHO definition of EBF and there was no difference among mothers in the different age groups.

### Complementary feeding practice by mother's age group

The mean of mothers’ intent/plan in the duration of continuing breast-feeding was 4·9 ± 3·1 months; younger aged mothers had less intent/plan in terms of duration of breast-feeding than older mothers (differences in mean −0·80, 95 % CI −1·58, −0·13; *P* = 0·046). Younger Saudi mothers generally introduced weaning food before older mothers by about 4 weeks (mean differences were −0·4, 95 % CI −0·71, −0·13; *P* = 0·031).

Regarding the introduction of different food types, the mean time for introducing a breast-milk substitute was at 3 ± 2·3 months; younger mothers introduced a breast-milk substitute earlier than older mothers and this difference was significant. This was followed by beverages other than milk at 3·3 ± 2·2 months, then ready-to-eat food, which was introduced at 5·6 ± 2·4 months. Furthermore, younger mothers introduced beverages other than milk and ready-to-eat food significantly earlier than older mothers, with differences in mean of −0·75; 95 % CI −1·33, −0·17; *P* = 0·011 and −0·85, 95 % CI −1·5, −0·22; *P* = 0·008, respectively. However, there were no significant differences between mothers’ age groups in terms of introducing homemade dishes and regular family food (Table 2).

**Table 2.** Complementary feeding practice by mother's age group

General feeding practices (infant's age per months)	All participants (*n* 247)		18–29 years (*n* 148)		30–45 years (*n* 99)					
	Mean	±sd	Mean	±sd	Mean	±sd	Coeff.	95 % CI		*P*^a^
First weaning food	4·1	1·4	3·8	1·2	4·3	1·4	−0·40	−0·71	−0·13	0·031
Time of first introducing										
Breast-milk substitute (formula, fresh or processed animal milk)	3·1	2·3	2·9	2·2	3·5	2·4	−0·65	−1·3	−0·50	0·034
Beverages other than milk	3·3	2·2	3·1	2·2	3·7	2·3	−0·75	−1·33	−0·17	0·011
Ready-to-eat food	5·6	2·4	5·3	2·0	6·1	2·8	−0·85	−1·5	−0·22	0·008
Infants’ food-homemade	4·5	1·2	4·4	1·2	4·6	1·4	−0·25	−0·58	0·10	0·149
Regular family food^b^	10·0	3·8	9·9	3·4	10·1	4·4	−0·37	−1·4	0·64	0·473

a Differences between mean younger mothers and older mothers in infant feeding practices were assessed by using a regression model with adjusting for occupation, mother education level, family income, mother BMI and infant gender.

b This has been asked for infants aged >6 months (*n* 152).

### Factors associated with complementary feeding age

Mothers’ age was significantly associated with the early introduction of complementary feeding before 17 weeks. Mothers of younger age (18–29 years) were 45 % less likely to provide complementary feeding at ≥17 weeks (OR 0·55; 95 % CI 0·31, 0·98; *P* = 0·041). In addition, mothers who had a caesarean section were 47 % more likely to introduce complementary feeding at <17 weeks (OR 0·47; 95 % CI 0·25, 0·88; *P* = 0·019). Mothers’ education, occupation and family income were not associated with mothers’ decision to introduce complementary feeding. However, mother's belief regarding the time to introduce complementary feeding, the infant's hunger, and promoting the infant's weight and sleep were all significant factors influencing the mothers’ decision about the time to introduce complementary feeding (Table 3).

**Table 3.** Factors associated with the introduction of complementary feeding by age (<17 weeks/≥17 weeks)

Factors	Total sample		Complementary feeding by infant age^a^					Adjusted^b^		
	(*n* 247)		<17 weeks (*n* 159)		≥17 weeks (*n* 88)			OR	95 % CI	*P*
	*n*	%	*n*	%	*n*	%	*P*			
Mother age group										
18–29 years	148	59·9	107	64·2	41	46·6				
30–45 years	99	40·1	52	32·7	47	53·4	0·001	0·55	0·31, 0·98	0·041
Mother education level										
High school and lower	61	24·7	39	24·5	22	25		1		
University	163	65·9	107	67·3	56	63·6		0·66	0·32, 1·4	
Post graduate	23	9·3	13	8·2	10	11·4	0·691	1·3	0·40, 3·99	0·278
Occupation										
Don't work	137	55·5	85	53·5	52	59·1		1·0		
Employed (part time)	43	17·4	36	22·6	7	7·9		0·35	0·20, 0·90	
Student	43	17·4	22	13·8	21	23·9		1·59	0·70, 3·20	
Employed (full time)	24	9·7	16	10·1	8	9·1	0·014	0·84	0·31, 2·30	0·060
Family income										
≤4·000	42	17·0	28	17·6	14	18·9		1·0		
5·000–11·999	120	48·6	74	46·5	46	52·3		1·43	0·65, 3·32	
12·000–19·999	59	23·8	38	23·9	21	23·9		1·10	0·43, 2·76	
≥20·000	26	10·5	19	11·9	7	7·9	0·720	0·72	0·22, 2·32	0·733
Delivery type										
Vaginal	155	62·7	92	57·9	63	71·6				
Caesarean section	92	37·3	67	42·1	25	28·4	0·033	0·47	0·25, 0·88	0·019
Mothers feeding practice reasons (yes)										
It is a good time to start complementary feeding	171	69·2	97	61·1	74	84·1	0·000	1·15	1·10, 1·40	0·000
I feel infant is hungry and needs sources other than milk	151	61·1	87	54·7	64	72·7	0·005	2·41	1·33, 4·30	0·004
Increase the infant's weight	85	34·4	46	28·9	39	44·3	0·015	1·10	1·10, 1·20	0·020
Promote sleep	83	33·6	47	29·6	36	40·9	0·071	1·34	0·94, 1·70	0·046
Breast-milk is not enough to meet the infant's nutritional needs	81	32·8	49	30·8	32	36·4	0·374	1·10	0·89, 1·30	0·387
Back to work	43	17·4	28	17·6	15	17·1	0·911	0·95	0·80, 1·10	0·347
Advice from parents and relatives	38	15·4	18	11·3	20	22·7	0·017	1·10	1·10, 1·20	0·009

a Univariate analyses using cross-tabulation and *χ*^2^ statistical tests to compare the differences in the proportion between mothers who complementary fed their infant <17 weeks and ≥17 weeks.

b Value is the odds ratio obtained from the logistic regression model. The model was adjusted for maternal education, occupation, family income, infant birth weight and gender.

### Maternal weaning practice and sources of information

**Fig. 1.**
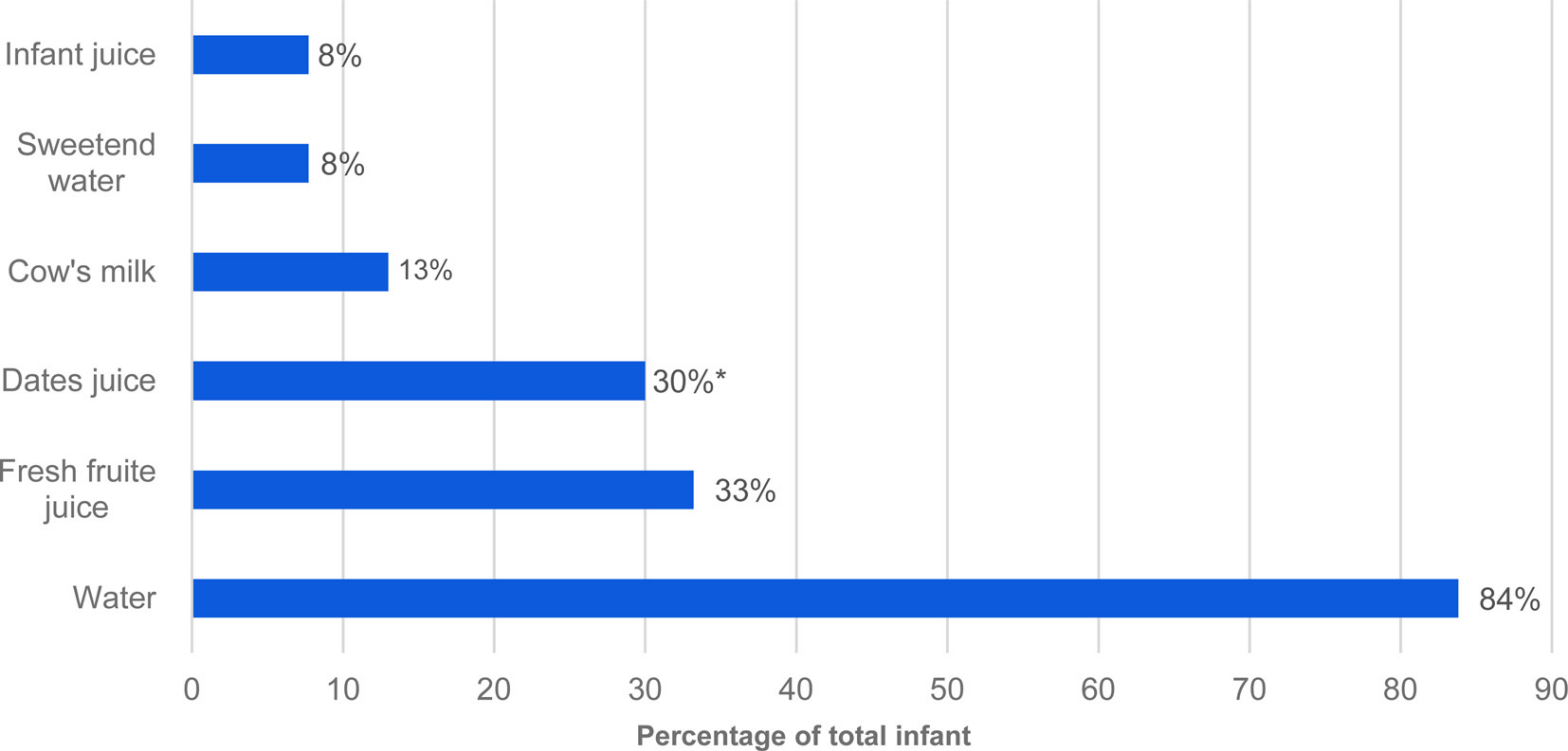
Fluids mothers usually introduce to infants. *Significant differences between the proportion of infants complementary fed at <17 weeks and at ≥17 weeks, using *χ*^2^ (significant at *P* = 0·033).

**Fig. 2.**
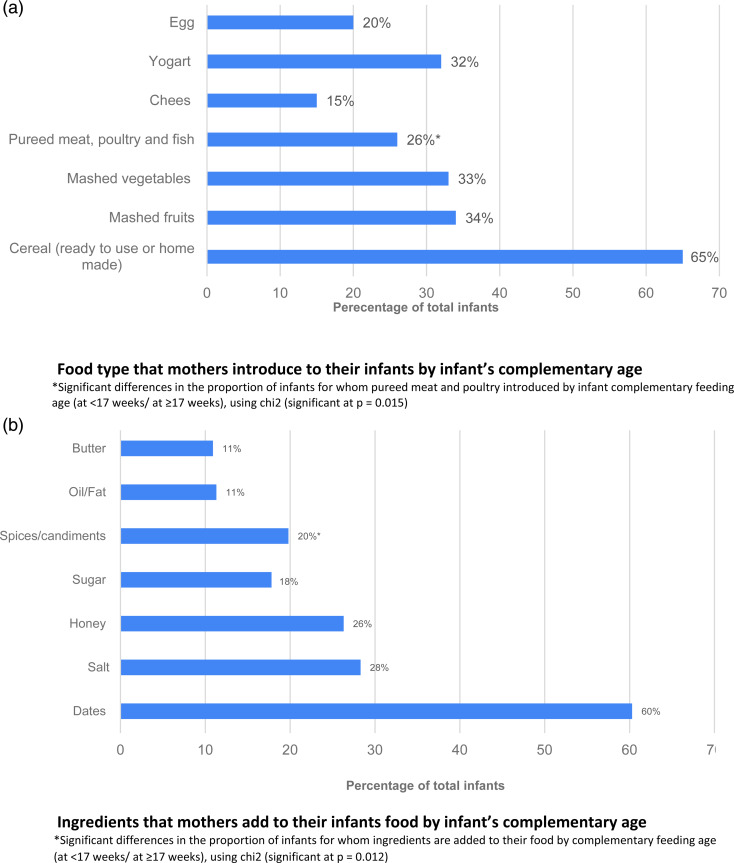
Food and ingredients that mothers introduce to their infants by complementary feeding age.

Fig. 1 shows fluids that mothers usually provide to their infants. Water was the most common fluid introduced to infants in total, then fresh fruit juice, and then date juice (84, 33 and 30 %, respectively). The proportion of infants introduced to date juice was higher among infants complementary fed at <17 weeks (34·6 %) compared to infants complementary fed ≥17 weeks (21·6 %); *P* = 0·033. Regarding foods that mothers initially introduced to their infants, cereal (ready to use or homemade) was the most common food item followed by mashed fruits and then mashed vegetables (65·2, 34·4 and 32·8 %, respectively). Pureed meat, poultry or fish was significantly different by infant complementary feeding age; 22 % of infants complementary fed at <17 weeks and 34 % of infants fed at ≥17 weeks; *P* = 0·015 (Fig. 2(a)). Furthermore, dates, salt, honey and sugar were the most common ingredients that Saudi mothers added to their infants’ weaning food, at 60, 28, 26 and 17·8 %, respectively. The proportion of mothers who added spices/condiments to their infants’ weaning food was significantly different by infant complementary feeding (28 % of infants complementary fed at <17 weeks and 15 % complementary fed at ≥17 weeks; *P* = 0·012) (Fig. 2(b)).

There are many sources of information that Saudi mothers use to get information about infant feeding practices. Online sources, family advice, consultant doctors and previous experience were the most common sources, at 61·6, 57·1, 51·4 and 46·2 %, respectively. The proportion of mothers who introduced complementary feeding before 17 weeks was significantly higher than mothers who introduced complementary feeding after 17 weeks based on online sources and following family advice (67 %, 54 %, significant at *P* = 0·04 and 63 %, 47 %; *P* = 0·013, respectively).

## Discussion

The present study demonstrates that there is a significant deviation from WHO recommendations among Saudi mothers in terms of infant feeding practices. Only 44·9 % of mothers breast-fed their infants after an hour of birth (EIBF) and 5·3 % of mothers with infants aged 6–12 months EBF. None of the infants aged 4–5 months received EBF. However, 92·7 % of mothers received EvBF. The average intent/plan of mothers to continue to breast-feed their infant was 4·9 (±3·1) months and younger mothers had significantly lower intent/plan than older mothers and provided a breast-milk substitute, beverages and ready-to-eat food at an earlier age than older mothers. A total of 64·3 % of infants were introduced to complementary feeding before completing 17 weeks. Maternal age group and mode of delivery were the only factors associated with the early introduction of complementary feeding. Most of the mothers attributed their feeding practices to their belief that ‘it is a good time’ and feeling that their infant was ‘hungry and in need of other sources of food’. Online sources and family advice were the top sources of information for the mothers in relation to infant feeding practices and there were significant differences between mothers’ age groups.

The WHO has set a global target of increasing EBF to at least 50 % for infants under 6 months by the year 2025^(28)^. However, European countries, the Eastern Mediterranean region (EMR) and upper middle-income countries are facing the greatest challenges in meeting these WHO targets. EMR has the lowest prevalence of EBF and experiences a decline from 43·7 % between 2000 and 2008 to 38·4 % between 2010 and 2018^(28)^. Recently, SA has witnessed a massive increase in women's participation in various work sectors, which may have led to changes in mothers’ feeding practices. Our findings confirm that there is a low rate of EBF practiced nationally, just as there is internationally. This study shows the lowest prevalence of EBF among infants below 6 months compared with 2018 when it was 16·3 %^(43)^ and 27·3 % before 18 years^(44)^. This is in line with the rate of EBF at 6 months in the UK, which remained at around 1 %^(45)^; on average, 13 % of infants are EBF during the first 6 months in the European region^(46)^.

In spite of the well-recognized benefit of breast-feeding, particularly in the first hour after childbirth, this study found that 44·9 % of mothers have EIBF. Similar to our finding, in 2019, the overall prevalence of EIBF in Saudi was 43·6 % and in Western regions, it was 45 %, which is considered fair^(9)^. This might be due to a lack of knowledge about its importance, as 82·6 % of Saudi mothers do not know of its importance. Dukuzumuremyi *et al.* found that 47·9 % of mothers disagreed about the importance of colostrum^(47)^. Another study found the rate of mothers’ intention to breast-feed in the first hour after childbirth was 13·9 %^(43)^, as reflected in our study which found a lack of knowledge among Saudi mothers regarding the valuable effect of breast-feeding in the first hour of an infant's life.

Regardless of EBF, the present study found that the average duration of ‘mother's intention’ to continue breast-feeding was also low (4·9 ± 3·1 months); the minimum was 1 month and the maximum was 12 months. This seems to suggest that the mean duration of breast-feeding has continued to decline in SA over time, especially these days due to the various developments that the kingdom is witnessing. It stood at 13·4 months in 1987 and declined to 8·5 months in 2010^(29)^. Lactation duration also dropped to 50 % at 6 months of an infant's age^(7)^. This is in line with a cohort study in Spain where it was found that the duration of breast-feeding was shorter among mothers who were younger^(48)^. Similarly, only 58·3 % of US infants were breast-fed at 6 months and mixed feeding practice in the first 2 days of life increased from 16·9 % in 2016 to 19·2 % in 2017^(49)^.

Although there is consensus on the WHO recommendations and other organisations such as the European Society for Paediatric Gastroenterology Hepatology and Nutrition^(32,50)^ agree that the earliest introduction of complementary feeding should not be done before 4 months or 17 weeks, 64 % of our sample introduced complementary feeding when the infant was <17 weeks and this was influenced significantly by the mother's age, with 45 % of younger mothers more likely to wean their infants at <17 weeks than older mothers. Higher rates (62·5 %) of early weaning practice have also been found in Tabouk city in SA and similar findings have been made in other Middle East countries. For example, in the United Arab Emirates, 65 % of infants were reported to have been introduced to solid foods at age 3 months^(51)^. In China, the median age for starting complementary feeding was found to be 4·5 months (95 % CI 4·4–4·6 months). Early weaning remains a concern not just in the Middle East but also internationally, as data from England and New Zealand show that 22·6 % of infants are prematurely introduced to solid foods at ≤ 12 weeks^(40)^.

Similar to the present study's findings, it has been found that increasing maternal age is associated with a high prevalence of breast-feeding and longer duration^(29,52)^, and younger aged mothers are more likely to practice early complementary feeding^(10,53)^. One cohort study found that an increase in mothers’ age added 2 % more breast-feeding time per year^(48)^. However, in 2015 studies based on Kuwait^(35)^ and the United Arab Emirates^(51)^, it was found that there was no positive association between mothers’ age and early complementary feeding. Factors associated with the early introduction of solid foods have been found to vary between different studies. For example, family income, occupation and mothers’ education have been found to be important factors linked with mothers’ early complementary feeding practices^(48,54)^. In a study among 1791 Saudi mothers in Riyadh, it was found that breast-feeding was higher among non-working mothers, those with lower family income, and those less educated^(55)^. Another study found that lower education, employment within 6 months post-birth, and low family income were factors associated with the early introduction of solid foods^(10)^. In line with our findings, a longitudinal study among 695 mothers found no association between the early introduction of food and family income^(56)^. Regarding mothers’ education level, our findings were consistent with previous findings for SA^(7)^, Kuwait^(35)^ and Turkey^(57)^. Furthermore, a 2019 Saudi study identified an enormous gap between EBF knowledge (95 % got more than 50 % of the questions right) and mothers’ feeding practices^(43)^.

In this research, 69·2 and 61 % of mothers changed their feeding practice as they thought it was ‘a good time to start’ and that the ‘infant is hungry and needs sources other than milk’. This was significantly different based on time of complementary feeding. Many studies have come to the same conclusion. In SA, one study found that 51·4 % of mothers believed that their infant was hungry and 26·6 % said that their child was old enough to be fed solid food^(8)^. In the MENA region, the common reasons given were ‘breastmilk is not naturally sufficient to feed three to four months old infants’ and the ‘child is old enough to have solid food’^(5)^. Similarly, Tarrant *et al.* found that later complementary feeding during the first 6 months was associated with later prediction of weaning time^(40)^.

Although the present study did not collect information on the amount and frequency of consumption of specific foods or fluids, fruit juice, date juice, baby cereal and pureed fruits were the most common foods and drinks provided to infants. Dates, honey and salt were the most common ingredients that mothers added to infant foods. Condiments were introduced significantly more among infants weaning at ≥17 weeks. This finding is in agreement with that of Tarrant *et al.*, which also found that with early weaning a high frequency of consumption of non-recommended weaning foods was provided to infants, drinking of sugary supplementary fluids and frequent addition of condiments to weaning food^(40)^. According to the Committee on Medical Aspects of Food Policy, unsweetened foods should be encouraged in preference to sugary food or drink among this age group^(58)^, as regular consumption of non-recommended foods during weaning practice increases the palatability of these foods and may affect long-term compliance with health eating guidelines^(50,58)^. Also, it has been suggested that it might lead to risk factors for being overweight and obese later in life^(59)^. The obesity rate among Saudi children and adolescents has doubled over a 10-year period compared with the WHO national prevalence^(60)^. Furthermore, consumption of diets rich in salt during infancy encourages a taste for salted foods and may contribute to an increased risk of blood pressure in later life^(61,62)^.

The present study found that ‘online sources’ and ‘family advice’ were the main sources of information about feeding practices among mothers. Another study found that younger mothers who introduced sub-optimal weaning practices used their maternal grandmother as their primary source of advice on infant feeding^(40)^. One study found that 65·3 % of mothers scored over 75 % when asked questions regarding the benefits of breast-feeding^(43)^. However, another study found that parents are not aware of the predicted harmful effects of early weaning and may perceive it to be helpful for their infant's health^(63)^.

Therefore, to address the high prevalence of sub-optimal feeding practices, public health initiatives are required that aim to improve the quality and quantity of infant feeding practices and strategies are needed to ensure compliance with weaning recommendations in Saudi Arabia, in particular among younger aged and first time mothers.

There may be a number of limitations related to this study. Firstly, the length of breast-feeding and feeding practice was reported by mothers and there may have been some recall bias due to the mother's social expectations, leading to over- or under-estimating of feeding practices^(64)^. Secondly, this was a cross-sectional study and so by its nature it prevented the determination of the direction of association. Also, no information regarding infant dietary intake portion size and frequency was collected; such information would advance our awareness of mothers’ feeding practices and sub-optimal weaning. On the other hand, results of this study shed light on many different aspects of mothers’ feeding practices. It has covered EBF, EIBF, EvBF and mothers’ practices in terms of introducing complementary food, as well as influencing factors, reasons and sources of information used for feeding practice. All of this information has the potential to provide policymakers in the public health sphere with a comprehensive understanding of Saudi mothers’ different feeding practices, particularly at this time after the COVID-19 pandemic and during the enormous developments that the kingdom is witnessing nowadays.

## Conclusion

The present study has shown that there is still a big gap between WHO feeding recommendations and what is actually occurring in SA, as EBF and duration of breast-feeding were poor and EIBF was fair. There is a high prevalence of sub-optimal weaning practices among Saudi mothers. Mothers’ age and mode of delivery was found to be an important factor influencing mothers’ feeding practice. Online sources and family advice were the most common sources of information for mothers regarding infant feeding. There is a need to conduct a cohort study at the country level to gain a broader understanding of mothers’ feeding practices and risk factors associated with them. The findings of this study can serve as baseline information for upcoming cohort/longitudinal studies. This study also highly emphasises the importance of initiating breast-feeding counselling and weaning advice for parents during the first year of an infant's life in order to increase EBF and compliance with healthy weaning guidelines.
