# Mothers’ feeding practices among infants (4–12 months) and associated factors: a cross-sectional study in Saudi Arabia – CORRIGENDUM

**DOI:** 10.1017/jns.2022.92

**Published:** 2022-10-17

**Authors:** Salwa Ali Albar

**Details of correction:** correct grant no. in acknowledgement

**Existing text:** “The Deanship of Scientific Research (DSR) at King Abdulaziz University (KAU), Jeddah, Saudi Arabia, has funded this Project Under grant no (G: 683-253-3443).”

**Corrected text should read:** “The Deanship of Scientific Research (DSR) at King Abdulaziz University (KAU), Jeddah, Saudi Arabia, has funded this Project Under grant no (G: 683-253-1443).”

